# Independent Processing of Stimulus-Stimulus and Stimulus-Response Conflicts

**DOI:** 10.1371/journal.pone.0089249

**Published:** 2014-02-18

**Authors:** Qi Li, Weizhi Nan, Kai Wang, Xun Liu

**Affiliations:** 1 Key Laboratory of Behavioral Science, Institute of Psychology, Chinese Academy of Sciences, Beijing, China; 2 University of Chinese Academy of Sciences, Beijing, China; University of Melbourne, Australia

## Abstract

The dimensional overlap (DO) model proposes distinct mechanisms for stimulus-stimulus (S-S) and stimulus-response (S-R) conflict effects. Many studies have examined the independence of S-S and S-R conflict effects in the color-word Stroop and Simon tasks. However, confounds exist between the distinction of DO (i.e., S-S dimensional overlap compared with S-R dimensional overlap) and the distinction of stimulus attributes (e.g., color compared with spatial location; semantic compared with nonsemantic information), which may hinder interpretation of the independence of S-S and S-R conflicts. A spatial Stroop (word) task and a spatial Stroop (arrow) task were combined with a Simon task in Experiments 1 and 2, respectively to eliminate these confounds of stimulus attributes. The results showed that S-S and S-R conflicts affected performance additively. There was no significant correlation across participants. These findings lend further support to independent processing of S-S and S-R conflicts as it is outlined in the taxonomy of DO.

## Introduction

Stimulus-response compatibility (SRC) refers to people performing more quickly and more accurately when mappings of stimuli to responses are congruent than when they are incongruent [1], [2]. Robust congruency effects have been observed when using a Stroop task [3], [4], a Simon task [5], [6] and other SRC tasks [7], [8]. In a typical Stroop task, people take longer and perform less accurately when identifying the print color of an incongruent color word compared with a congruent color word [3]. In a typical Simon task, people make slower and less accurate responses when the stimulus location does not correspond to the location of the assigned response than when it does [5], [6].

The Dimensional Overlap (DO) model, which was initially proposed as a taxonomy of SRC phenomena, includes a variety of SRC effects in one unified theory [9]. According to this model, S-R ensembles have a task-relevant stimulus dimension, a task-irrelevant stimulus dimension and a response dimension, and DO can occur independently between any of these two components. For example, in a manual Stroop task that employs arbitrarily mapped manual button press responses, there is an S-S conflict due to the overlap between only the relevant and irrelevant stimulus [10]. In a Simon task, S-R conflict is caused by the overlap between the irrelevant stimulus attribute and the response [6], [11]. These two effects can affect performance independently [12], [13].

According to the DO model, there is a distinction in resolving conflicts that arise from S-S and S-R incongruency. Several studies have factorially combined the color-word Stroop and Simon tasks into a single experimental protocol to explore the independence of S-S and S-R conflict effects by presenting a colored word on the left or right of the screen [13]–[15].

Evidence from behavioral studies supports the additivity of the congruency effects that result from S-S and S-R conflicts, which indicates independent processing of these conflicts [14], [15]. Researchers have additionally taken advantage of the conflict adaptation effects (which refer to the reduction in the SRC effect after processing of an incongruent compared with a congruent stimulus) to provide additional support for the possible independence of S-S and S-R conflicts [16]. Some studies using a combined-conflict paradigm (involving the Stroop/Flanker and Simon tasks) reported that the conflict adaptation effect was only observed when the same type of conflict (i.e., both conflicts were either S-S or S-R) was repeated, but not when the type of conflict was alternated (one was an S-S conflict and the other was an S-R conflict, and vice versa) across trials. These findings suggest that cognitive control mechanisms flexibly adapt to S-S and S-R conflicts by modulating information processing in ways that specifically address the source of conflict and that the control resources underlying S-S and S-R conflicts are independent of each other [10], [17], [18]. Researchers have also examined the effect of temporal distribution characteristics on the dissociation between S-S and S-R conflict effects. They reported that the S-S conflict effect increased and the S-R conflict effect decreased as the delay (SOA, Stimulus Onset Asynchrony) between the presentation of the irrelevant and relevant stimuli was increased [13]. Kornblum et al. (1999) therefore suggested that the different time courses of S-S and S-R conflict effects might be due to their conflict processing being serial, and modification of the SOA value directly determined different activation levels of the irrelevant stimulus at the onset of the stimulus processing stage and the response selection stage. Many neuroimaging studies have additionally revealed that distinct neural mechanisms are associated with S-S and S-R conflicts. Resolution of S-R conflicts activated the premotor cortex, the rostral portion of the dorsal cingulate cortex, and the posterior cingulate cortex. In contrast, resolution of S-S conflicts activated the parietal cortex and the caudal portion of the dorsal cingulate cortex [10], [19], [20]. A recent EEG study reported that S-S conflicts modulated the N2 and early P3 components and S-R conflicts modulated the late P3b component, which suggests that resolution of S-S and S-R conflicts may involve discrete temporal processing [20].

However, results from previous studies that used a combined S-S (color-word Stroop task or color-dot Flanker task) and S-R (Simon task) conflict paradigm to examine possible independent processing of S-S and S-R conflicts were not conclusive [5], [10], [13], [14], [20], [21]. It should be noted that both the color-word Stroop task and the color-dot flanker task involve the conflict of color information. Specifically, the former conflict was between ink colors and color words, and the latter conflict was between target color and flanker colors. However, the Simon task involves the conflict of spatial information between stimulus locations and responses. Behavioral and neuroimaging studies reported that interference in the color-word Stroop task resulted from semantic competition [22]–[24] but interference in Simon task came from nonsemantic conflict [5]. It therefore has not been determined whether the different patterns of these tasks were affected by the distinction of S-S and S-R conflicts or by differences in stimulus attributes, specifically “color compared with spatial” or “semantic compared with nonsemantic” information.

To address these limitations, we designed a factorial combination of SRC tasks to examine the independence of S-S and S-R conflict effects in two experiments in which a spatial Stroop (word/arrow) task and a Simon task were combined to rule out the contribution of stimulus attributes. In Experiment 1, we combined the spatial (word) Stroop task and Simon task in which a spatial word (“up” or “down”) was presented at one of the nine positions on a 3×3 lattice. Specifically, we used a spatial-word Stroop task that involves the conflict of spatial information between word locations (i.e., “top” or “bottom”) and spatial words (i.e., “up” or “down”) and a Simon task that involves the conflict of spatial information between word locations (i.e., “left” or “right”) and responses. Therefore, both S-S and S-R conflicts were related to spatial information. This design allowed us to rule out the contribution of color, which was usually confounded with spatial information in previous studies comparing color-word Stroop and Simon effects. However, the S-S conflict in the spatial (word) Stroop task was semantic but the S-R conflict in the Simon task was nonsemantic. We combined the spatial (arrow) Stroop task and the Simon task in Experiment 2 to eliminate this confound between semantic and nonsemantic information. We presented an “upward” or a “downward” arrow at one of the nine positions on a 3×3 lattice. The spatial-arrow Stroop task involves the conflict of nonsemantic spatial information between arrow locations (i.e., “top” or “bottom”) and arrow orientation (i.e., “upward” or “downward”), and the Simon task involves the conflict of nonsemantic spatial information between arrow locations (i.e., “left” or “right”) and responses. Therefore, both the S-S conflicts that resulted from the spatial (arrow) Stroop task and the S-R conflicts that resulted from the Simon task were related to nonsemantic spatial information. This further eliminated differences in stimulus attributes as a confound. We hypothesized that if S-S and S-R conflicts are processed in parallel by distinct mechanisms, then any SRC effects that arise from S-S and S-R conflicts would be additive. Otherwise, if processing of S-S conflicts and processing of S-R conflicts share a common pathway, the two types of conflicts would interact with each other and would show a sub-additive or super-additive effect when both S-S and S-R conflicts are present [25].

## Materials and Methods

### Participants

All participants reported that they had no neurological or psychiatric history. Each participant voluntarily enrolled and signed an informed consent statement prior to the experiments. Thirty university students (19–26 years old, average of 23±.35 years old, 15 men) participated in both experiments within one session. The order of the experiments was counterbalanced across participants. This research was approved by the Institutional Review Board of the Institute of Psychology, Chinese Academy of Sciences.

### Tasks and stimulus materials

**Figure 1:**
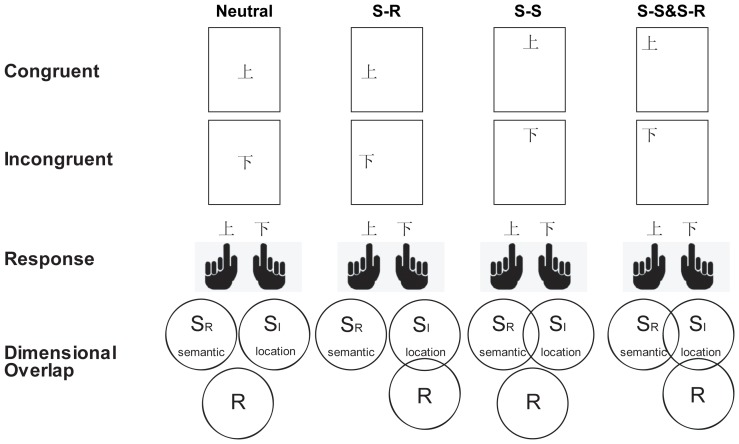
Experimental design for the spatial Stroop (word)-Simon task. S_R_ - task-relevant stimulus dimension; S_I_ - task-irrelevant stimulus dimension; R - response dimension. “Congruent” and “Incongruent” labels do not apply to Neutral trials. The Chinese words, “?” and ”?”, mean “up” and “down”, respectively.

**Figure 2:**
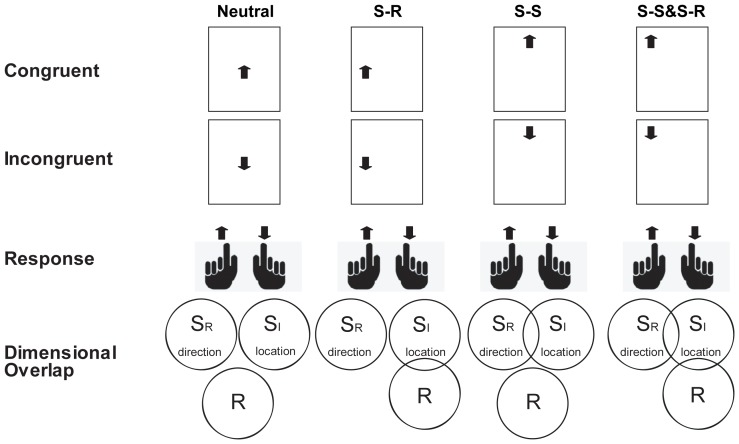
Experimental design for the spatial Stroop (arrow)-Simon task. Labels and legends are the same as in Figure 1.

A modified spatial Stroop (word/arrow)-Simon task [26] was used in Experiments 1 and 2. During training, a stimulus (the word “up” or “down” in Experiment 1 or an upward or downward arrow in Experiment 2) was presented at the center of the screen. Half of the participants responded to the word “up” or “down” (in Experiment 1) and the upward or downward arrow (in Experiment 2) with their left and right index fingers, respectively. The mapping was counterbalanced for the other half of the participants in both experiments. During testing, a word or arrow was presented at one of the nine possible locations in the 3×3 lattice (see Figures 1 and 2).

Participants were required to ignore the location of the stimulus and respond only to the meaning of the word and the direction of the arrow according to the rules on which they had been trained in Experiments 1 and 2. Trials were classified into different conditions based on the presence and nature of the conflict (e.g., S-S or S-R). For example, when the target was presented in the center of the screen, the trial was considered neutral (SSNSRN) because the location of the target did not overlap with either the direction (in meaning) of the target or the response to the target. In some trials (data represented in the second column of the figures), the target was presented on the horizontal axes of the screen and represented dimensional overlap between target locations (task-irrelevant dimension) and responses (SSNSRC and SSNSRI). Specifically, the left or right location of the target overlapped with the left or right response but not with the task-relevant dimension of the stimulus (i.e., the word “up” or “down” in Experiment 1 and an upward or downward arrow in Experiment 2). In still other trials (data represented in the third column of the figures), the target was presented on the vertical axes of the screen and represented dimensional overlap between task-relevant and task-irrelevant dimensions (SSCSRN and SSISRN). Specifically, the location of the target (i.e., top or bottom) overlapped with the direction of the target (i.e., the word “up” or “down” in Experiment 1 and an upward or downward arrow in Experiment 2) but not with the left or right response. In the fourth type of trial (data represented in the fourth column of the figures), the target was presented at one of the four corners of the screen and illustrated dimensional overlaps of both S-S (task-irrelevant and task-relevant dimensions) and S-R (task-irrelevant and response dimensions) ensembles, which were independently manipulated (SSCSRC, SSCSRI, SSISRC, and SSISRI). Each participant completed six runs of 90 trials each. Nine types of stimuli were equally and randomly presented in each run. In each trial, following a fixation of 200±100 ms, a word or an arrow was shown for 600 ms. The trial ended with another fixation of 1500±100 ms.

## Results

Reaction times (RTs) and error rates (ERs) were compared across conditions with repeated-measure ANOVAs in all experiments (see Table1). The significance level was set at *α*<.05 for all ANOVAs. The effects of interest were summarized below.

**Table 1:** Reaction times and error rates for the spatial Stroop (word/arrow)-Simon task.

	Experiment 1		Experiment 2	
Condition	RT(ms)	ER(%)	RT(ms)	ER(%)
	M(SD)	M(SD)	M(SD)	M(SD)
SSNSRN	458(50)	3(4)	484(76)	5(4)
SSCSRN	458(53)	2(3)	478(80)	4(3)
SSISRN	478(49)	6(5)	511(73)	6(6)
SSNSRC	449(49)	2(2)	469(64)	2(2)
SSNSRI	470(47)	6(5)	506(81)	7(7)
SSCSRC	448(49)	2(2)	467(60)	2(2)
SSCSRI	472(51)	4(5)	500(77)	6(4)
SSISRC	471(50)	4(4)	502(66)	4(4)
SSISRI	494(50)	8(8)	530(81)	11(9)

Notes: Means (M) and standard deviations (SD) of the reaction times (RT) and error rates (ER) for the spatial Stroop (word)-Simon task (Experiment 1), and spatial Stroop (arrow)-Simon task (Experiment 2).

Nine conditions can be considered as a factorial combination of S-S (congruent, neutral, incongruent) and S-R (congruent, neutral, incongruent) congruency. One 3×3 repeated-measure ANOVA was conducted to examine the main effects of S-S and S-R congruency and their interaction on RTs, and another was conducted to examine the main effects of S-S and S-R congruency and their interaction on ERs.

**Figure 3:**
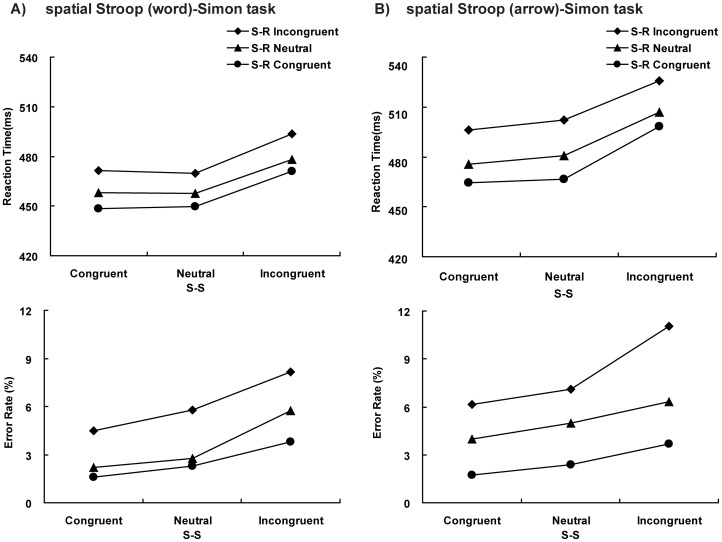
Reaction times and error rates of two tasks. (A) the spatial Stroop (word)-Simon task, (B) the spatial Stroop (arrow)-Simon task.

In Experiment 1, RTs showed significant main effects for both S-S and S-R congruency [S-S, *F*(2,58) = 109.16, *p*<.001, η_p_^2^ = .79; S-R, *F*(2,58) = 75.50, *p*<.001, η_p_^2^ = .72]. The interaction was not significant [*F*(4,116) = .44, *p*>.05, η_p_^2^ = .02], indicating that there was an additive effect between S-S and S-R congruency. ERs showed significant main effects for both S-S and S-R congruency [S-S, *F*(2,58) = 20.72, *p*<.001, η_p_^2^ = .42; S-R, *F*(2,58) = 13.01, *p*<.001, η_p_^2^ = .31]. The interaction was not significant [*F*(4,116) = 1.02, *p*>.05, η_p_^2^ = .03], indicating that there was an additive effect between S-S and S-R congruency. ERs were positively associated with RTs across conditions, which rules out a speed–accuracy trade-off effect (see Figure 3A).

Patterns similar to those of Experiment 1 were found in Experiment 2. RTs showed significant main effects for both S-S and S-R congruency [S-S, *F*(2,58) = 118.49, *p*<.001, η_p_^2^ = .80; S-R, *F*(2,58) = 43.64, *p*<.001, η_p_^2^ = .60]. The interaction was not significant [*F*(4,116) = 1.00, *p*>.05, η_p_^2^ = .03]. ERs showed significant main effects for both S-S and S-R congruency [S-S, *F*(2,58) = 16.04, *p*<.001, η_p_^2^ = .36; S-R, *F*(2,58) = 30.54, *p*<.001, η_p_^2^ = .51]. The interaction was not significant [*F*(4,116) = 2.12, *p*>.05, η_p_^2^ = .07]. ERs were positively associated with RTs across conditions (see Figure 3B).

Correlation analyses across participants in both experiments revealed that single effects of S-S and S-R conflicts were not significantly correlated [Experiment 1: *r*(28) = −.36, Experiment 2: *r*(28) = −.05, *ps*>.05], which implies that each effect is relatively independent.

## Discussion

The results of both experiments showed that task performance was independently affected by S-S and S-R conflicts, even though both types of conflicts were related to spatial attributes. This design eliminated the confound of different stimulus attributes in the interpretation of S-S and S-R independence. These novel findings support and enhance the taxonomy of the DO model.

Earlier studies that compared the color-word Stroop task and the Simon task indicated that S-S and S-R conflicts might be processed independently [10], [14], [15], [21], [26]. However, the interpretation of this distinction may be confounded by differences in stimulus attributes (e.g., color compared with spatial location). We combined a spatial Stroop (word) task and a Simon task in Experiment 1 to eliminate the possible confound between stimulus attributes (e.g., color compared with spatial location) that made interpretation of the results of previous studies difficult. We found that the effects of S-S and S-R conflicts were additive, which indicates that the processing of S-S conflict and the processing of S-R conflict are independent.

Previous behavioral studies have also indicated that the interference in a color-word Stroop task arose from semantic competition [22]–[24], [27] but that interference in a Simon task originated from nonsemantic conflict [5]. Neuroimaging research showed that a Stroop task (involving semantic conflict) and a Simon task (involving spatial conflict) recruited either distinct neural networks or different sites of a single network [21]. In Experiment 2, we combined the spatial Stroop (arrow) task and a Simon task to eliminate a confound between other stimulus attributes (e.g., semantic compared with nonsemantic information) that had been found in previous studies. We found that the effects of S-S and S-R conflicts were still additive, which indicates that S-S conflict processing and S-R conflict processing are independent.

One previous study that also examined a combination of the spatial Stroop and Simon tasks found an interaction of S-S and S-R conflict effects [28]. This pattern appears to be contrary to the present findings and would argue against the independence of S-S and S-R conflict processing. However, there was a critical issue in the above-mentioned study that involves the interaction of S-S and S-R conflict effects. The researchers inserted a word-alone control condition for one-third of the trials to ensure a strong Stroop effect. In this condition, the participants were instructed to respond to word meaning (“low” or “high”) instead of the location of the word (above or below three plus signs). Stimuli were presented on the left or right of fixation point, and participants responded with a left or right button press. This strategy was problematic because it introduced task-switching in different conditions. Specifically, participants in the Stroop congruent/incongruent trials were asked to ignore the word meaning but in control trials they were asked to attend to and respond to word meaning. Indeed, control trials increased the interference of word meaning in the incongruent Stroop trials, such that the effect size of the Stroop effect was more than four times that of the Simon effect. Another problem with this cited study is that task-switching itself relied on cognitive control resources [29], and task-switching costs were likely to interact with conflict processing [30]. Therefore, studies involving a task-switching design are not well suited to determining the specificity of conflict-driven control mechanisms [16].

Further, both of the previous experiments that combined a spatial Stroop task with a Simon task found that S-S and S-R conflict effects on RTs were not significantly correlated with each other. This further implied that although these conflicts both resulted from the same spatial inconsistency, a person's ability to resolve an S-S conflict was not related to his/her ability to resolve an S-R conflict. This finding supports independent mechanisms of S-S and S-R conflict processing. Our findings were consistent with previous studies comparing the Stroop and Simon tasks, which also showed that SRC effects caused by S-S and S-R conflicts were not correlated [14], [21]. As Egner and colleagues suggested, it is unlikely that people recruit a single domain-general conflict resolution network to process all types of conflicts. There may instead be several independent conflict detection and/or control nodes that operate in parallel to resolve domain-specific conflicts [10], [16].

The novel aspect of the current study was our expanding the spatial Stroop-Simon paradigm to examine independent processing of S-S and S-R conflicts. A similar spatial Stroop-Simon paradigm, initially reported by Liu and colleagues [26], was designed to eliminate differences in stimulus attributes and to determine differences in the neural substrates activated by the nature of the S-S or S-R conflict. Other researchers have subsequently conducted a series of studies using the spatial Stroop-Simon paradigm [18], [31]–[33]. For example, Funes and colleagues argued that there is a dissociation between a proportion-congruent effect and a conflict-adaptation effect [32], [33]. Our study found that S-S and S-R conflict processing differed in temporal and spectral dynamics [31]. However, in previous studies incorporating the spatial Stroop-Simon paradigm, an arrow was presented at only one of the four possible locations (left, right, top, bottom), and S-S and S-R conflicts were induced separately in different trials. Such control can rule out the contribution of stimulus attributes but cannot use the additive-factor method to test for separate control systems for S-S and S-R conflicts. In contrast to previous studies, we developed a novel experimental design in which an arrow was presented at one of nine positions on a 3×3 lattice. S-S and S-R conflicts could therefore occur separately in different trials (when the arrow was at one of the four vertical/horizontal positions) and also simultaneously during the same trial (when the arrow was at one of the four corners). This design enabled us to use Sternberg's additive factor method to test whether S-S and S-R conflicts were processed independently.

Our current research and previous studies taken together provide strong support for the independent processing of S-S and S-R conflicts as proposed by the DO model and for the dissociable processing of these conflicts that may recruit distinct control mechanisms [10], [20]. There may also be a common control mechanism for resolving different conflicts [21], [26], [34]–[37]. For example, Jiang & Egner, employing neural pattern classifiers to quantify the modularity of conflict-control mechanisms, found that there were both domain-specific and domain-general processors in the human brain that carried the discriminating information that allowed the classification [34]. Complete understanding of these systems requires further research that combines additional behavioral measures (e.g., conflict adaptation effect) and such techniques as neuropsychological testing of patients with brain lesions, fMRI, EEG, and computational modeling to elucidate psychophysical properties, functional localization, temporal components of these conflicts and their resolution, and integration of brain networks [10], [14], [20], [38], [39].
